# Energy Density of New Food Products Targeted to Children

**DOI:** 10.3390/nu12082242

**Published:** 2020-07-27

**Authors:** Danielle J. Azzopardi, Kathleen E. Lacy, Julie L. Woods

**Affiliations:** 1 Deakin University, School of Exercise and Nutrition Sciences, Geelong, VIC 3220, Australia; dazzopar@deakin.edu.au; 2 Deakin University, Institute for Physical Activity and Nutrition (IPAN), School of Exercise and Nutrition Sciences, Geelong, VIC 3220, Australia; j.woods@deakin.edu.au

**Keywords:** energy density, health star rating, children, food supply, front-of-pack label, discretionary

## Abstract

High dietary energy density (ED) is linked to childhood obesity and poor diet quality. The Australian Health Star Rating (HSR) system aims to assist consumers in making healthful food choices. This cross-sectional study used 2014–2018 data from the Mintel Global New Products Database to describe the ED of new food products targeted to children (5–12 years) released after the introduction of HSR and examine relationships between ED and HSR. Products were categorised by ED (low < 630 kJ/100 g, medium 630–950 kJ/100 g, high > 950 kJ/100 g) and HSR (no, HSR < 2.5 low, HSR ≥ 2.5 high). Non-parametric statistics were used to examine ED and HSR. A total of 548 products targeted children: 21% low, 5% medium, 74% high ED. One hundred products displayed an HSR: 24% low, 76% high; 53 products with both high HSR and ED. The EDs of products differed by HSR (*p* < 0.05), but both group’s medians (HSR < 2.5: 1850 kJ/100 g, HSR ≥ 2.5: 1507 kJ/100 g) were high. A high proportion of new products had a high ED, and the HSR of these foods did not consistently discriminate between ED levels, particularly for high ED foods. Policies to promote lower ED foods and better alignment between ED and HSR may improve childhood obesity and diet quality.

## 1. Introduction

Childhood overweight and obesity are global concerns. The worldwide prevalence of overweight and obesity in children and adolescents is just over 18% [1]. In Australia, at least one in four children and adolescents aged 5–17 years are currently considered overweight or obese [2]. Measures of overweight, obesity, and adiposity are positively associated with dietary energy density [3,4,5].

The energy density (ED) of a food is defined as the amount of energy in a specific weight of that food and is usually expressed as kilojoules per 100 g (kJ/100 g). The macronutrient composition and moisture content of the food determine its ED, with foods higher in fat tending to have higher EDs than other foods and water-rich foods tending to have lower EDs than other foods. Food energy density is potentially modifiable by adjusting the macronutrient and/or moisture content of foods. Multiple within-subject crossover design experimental feeding studies have demonstrated that lowering the ED of foods, while maintaining their palatability, reduces children’s energy intake (EI) [6,7,8]. Dietary energy density can be reduced by adjusting food energy density, incorporating more foods that are lower in energy density into the diet or reducing consumption of energy-dense foods. In children, decreasing the ED of the diet is a way to prevent overconsumption of energy without reducing EI below the child’s current needs [7] and could contribute to a reduction in rates of childhood obesity.

Diets that are lower in ED tend to be of higher quality [9,10,11] and more in line with dietary guidelines [9]. They tend to include plenty of vegetables, fruit, wholegrain cereals, low-fat dairy, lean sources of protein and healthy oils [12]. There is strong evidence that higher ED diets are of lower quality [9,10,11]. Studies involving children and adolescents in several countries have shown dietary ED to be positively associated with the consumption and availability of discretionary foods high in sugar and fat and inversely associated with the consumption and availability of fruit, vegetables, protein and fibre [13,14,15,16].

Several population-based surveys have found that Australian children regularly consume discretionary foods [17,18,19]. The Australian National Health Survey found that just under 40% of the total energy consumed by 4- to 13-year-old children came from discretionary foods [19]. Children in this age group have considerable input into the food products purchased for them by their carers, initially through “pester power” [20] and then through a more collaborative decision-making process [21]. Understanding the retail food supply targeting this age group is important for developing strategies to improve children’s dietary intakes.

The Health Star Rating (HSR) system was introduced in Australia in 2014 as a voluntary, front-of-pack label to assist consumers in making healthy food choices in a discretionary food-flooded environment. This system rates the overall healthiness of a product on a scale from a half to five stars, with a greater number of stars indicating a healthier product [22]. The number of stars is calculated based on an algorithm, which scores ‘negative’ nutrients (energy, saturated fat, total sugar and salt) and ‘positive’ attributes (fruit, vegetable, legume and nut content and, in some cases, protein and fibre content). The number of stars a product receives should increase as its ED decreases, giving this system the potential of assisting consumers in choosing lower ED options. Being a voluntary system for food manufacturers, only 40.7% had taken it up in 2019 [23]. It is evident that manufacturers are selectively applying the HSR to foods, which score ≥ 3.0 stars (i.e., healthier choices), and have been reluctant to display it on foods with lower star scores, although supermarket own brands have applied it across all products regardless of the score [24].

Despite the current knowledge of dietary ED and its links with increased EI, lower diet quality and obesity, no studies have examined the ED of new food products targeted to children entering the retail food market in Australia, either long-term or since the introduction of the HSR system. Advertising and supermarkets target children and promote the consumption of discretionary foods [25,26,27]. The high ED of such foods results in a greater likelihood of excess EI and overweight and obesity. Examining the ED and HSR of these products is vital to provide a greater understanding of the food supply and the potential of the HSR system in being able to distinguish between foods with high and low ED. The results of this study could potentially be used to advocate for change in the HSR system, which, in turn, may influence food manufacturers to release products with lower ED.

The aims of this study were to:

1. Describe the ED of new food products targeted to children that have entered the Australian retail food market since the introduction of the HSR system in 2014.
2. Examine the relationship between the ED and the HSR of new food products targeted to children that have entered the Australian retail food market and display an HSR.
3. Examine the relationship between core and discretionary products, ED and the HSR of new food products targeted to children that have entered the Australian retail food market and display an HSR.

## 2. Materials and Methods

This cross-sectional study examined the ED and HSR, where available, of all new products targeted to children and launched in Australia from 27th June 2014 to 27th June 2018 recorded in the Mintel Global New Products Database [28].

### 2.1. The Mintel Global New Products Database

The Mintel Global New Products Database (GNPD) is an online database of consumer-packaged goods from 62 countries, created and maintained by Mintel, a private international market research company [28]. A network of trained Mintel shoppers frequently monitors the release of new products and updates the database at least monthly. The database captures more than 80 fields of information per item for 17 distinct categories of foods: Baby Food, Bakery, Breakfast Cereals, Chocolate Confectionery, Dairy, Desserts and Ice-Creams, Fruits and Vegetables, Meals and Meal Centres, Processed Fish, Meat and Eggs, Sauces and Seasonings, Savoury Spreads, Side Dishes, Snacks, Soups, Sweets and Gum, Sweet Spreads and Sweeteners and Sugar. The dataset consists mainly of packaged foods and does not generally include fresh, non-processed single foods, such as fresh fruit and/or vegetables.

GNPD fields of information include nutrient data, packaging format, claims made and manufacturing details. The GNPD records if a product is targeted to a particular demographic, namely, babies and toddlers (0–4 years), children (5–12 years), teenagers (13–17 years), females, males and seniors (aged 55+ years). The present study used the children (aged 5–12 years) demographic category. As of 27th June 2018, the database listed 62,066 foods and beverages released in Australia since its launch in 1998. A total of 2683 of these are included in the children 5–12 years demographic category, with 579 products added under this demographic since June 2014 [28].

#### 2.1.1. Search for Products in Demographic 5–12 Years

The GNPD was searched using filters that restricted results from June 2014 to June 2018 in Australia, to foods (not beverages) and for children aged 5–12 years. The GNPD defines this demographic category as foods designed for consumption by children and, more specifically, products which are “also dependent on presentation and format, such as child-inspired graphics like cartoon characters, bright colours and/or pictures of children, or particular language like ‘great in lunch boxes’ [29]. Data from all 17 GNPD food categories were used in this study; however, some sub-categories were excluded. Beverages were excluded because the grouping of beverages and foods together when calculating ED complicates the interpretation of the results, as beverages are relatively low in ED due to their high water content and can have a substantial impact on overall ED values [30].

#### 2.1.2. Additional Product Searches

To ensure no products were missed, further searches were performed without the demographic filter but with the addition of relevant keywords, such as ‘children’, in the product description. These searches also used filters that restricted results from June 2014 to June 2018 in Australia.

#### 2.1.3. Data Extraction

In this study, data from 10 of the 80 fields available for each product were extracted from the GNPD: date published, company, brand, product name, category, sub-category, energy (kJ/100 g), demographic, packaging pictures and ingredients list. Company and brand fields were extracted to help exclude duplicates and identify products that display an HSR. Packaging images and descriptions of each product were extracted in order to determine the presence of an HSR, as the GNPD does not routinely include information about HSR. Data were downloaded into Microsoft Excel for analysis.

Sorting was used to remove duplicates from multiple searches. Seasonal products, such as Halloween confectionary and Easter chocolates, were also removed as these products are not available all year and so do not make up the typical range of food items available to children. It is possible for a product to lie within multiple demographics; for example, Bellamy’s Organic Apple Snacks are listed with both the 0–4 years and 5–12 years demographics. These records were retained, even if the food category was Baby Food. The description and packaging images for such products were examined, and the product was removed if determined unsuitable (e.g. supplement or formula drinks/foods, such as Ensure). Where a product was a variety pack, that is, two or more flavour varieties of the same or similar food in the one pack, the record was duplicated for each variety. The overall total of products was 548.

#### 2.1.4. Data Cleaning

Data were checked for accuracy and completeness, and a total of 23 records were found to be missing the value for ED (kJ/100 g). Thirteen of these were variety packs, and the missing data were found on the nutrition information panels from the product images. For 9 of the remaining records, missing data were retrieved from similar products of the same brand (*n* = 2) or different brands (*n* = 7) in the GNPD. Missing data for the final record (Tic-Tac) were obtained from the product website.

### 2.2. Determination of Energy Density Category, HSR Presence and Core or Discretionary Classification

Products were categorised into one of three ED categories (low: <630 kJ/100 g; medium: 630–950 kJ/100 g; high: >950 kJ/100 g), according to those defined by the World Cancer Research Fund [31]. Whether a product had an HSR and the number of stars it had was determined by examining packaging images from the GNPD and were added to the relevant record. Each product with an HSR was then classified as discretionary or core, according to the Discretionary Food List produced by the Australian Bureau of Statistics (ABS) in the Australian Health Survey User Guide [32].

### 2.3. Statistical Analysis

All statistical analyses were conducted in IBM SPSS Statistics version 23 (IBM, St Leonards, NSW, Australia). The ED data were not normally distributed, and so medians and interquartile ranges (IQRs) were reported and used for analysis. Descriptive statistics (frequency, median, IQR, minimum and maximum) for EDs were calculated for each food category, products without an HSR, products with an HSR, products with a low HSR (<2.5 stars) and products with a high HSR (≥2.5 stars). Mann-Whitney U tests were performed to compare the EDs of foods with an HSR to those without, foods that had low (<2.5) and high (≥2.5) HSRs and foods that were classified as core or discretionary. A Chi-square test was performed to compare the proportions of low, medium and high ED products within the groups of products with and without an HSR. The proportion of products with <2.5 stars and ≥2.5 stars in each of the three ED categories was determined, but inferential statistics could not be performed due to violations of assumptions for non-parametric statistical tests.

### 2.4. Ethics

This study did not include an animal or human participants or existing data collected from them and so, in accordance with Australia’s National Statement of Ethical Conduct in Human Research [33], is deemed negligible risk and did not require ethical review.

## 3. Results

### 3.1. All Products: GNPD Food Category Distributions and Energy Densities

The 548 food products targeted to children released into the Australian market between 27th June 2014 and 27th June 2018, were from 14 of the 17 food categories in the GNPD. No products were found from the Sauces and Seasonings, Soup and Sweeteners and Sugar food categories. The greatest proportion of foods (30.7%) was from the Snacks category, and almost half (49.3%) were from the Snacks and Bakery categories combined. The additional inclusion of the discretionary categories Chocolate and Confectionary, Desserts and Ice Cream and Sugar and Gum Confectionery represented 76.5% of the entire sample.

The EDs for the sample ranged from 6 kJ/100 g to 2556 kJ/100 g (Table 1). Aside from Fruit and Vegetables (138 kJ/100 g), the categories with the lowest median EDs were Dairy (377 kJ/100 g) and Desserts and Ice Cream (409 kJ/100 g). Nine of the 14 categories had median EDs that were considered high (i.e., >950 kJ/100 g), although three of these categories had low numbers of foods (*n* < 3). There were 117 (21.4%) products that were categorised as low ED, 28 (5.1%) as medium ED, with the vast majority categorised as high ED (*n* = 403, 73.5%). In particular, more than 86% of the Bakery, Breakfast Cereals and Snacks items had high EDs, and more than 98% of the Chocolate Confectionery and Sugar and Gum Confectionery items had high EDs.

### 3.2. Products With and Without an HSR: GNPD Food Category Distributions and Energy Densities

One hundred (18.2%) of the 548 products in the sample displayed an HSR on the packaging (Table 2). Nine out of the 14 categories of foods targeted to children contained products that displayed an HSR. Three categories (Bakery, Breakfast Cereals and Snacks) made up 80% of all items displaying an HSR and had high median ED. All of the Bakery and Breakfast Cereals items with HSRs had high EDs. Although the median EDs of the group of foods without an HSR and the group of foods with an HSR were similar (1490 kJ/100 g and 1594 kJ/100 g, respectively), the variability of the EDs for the group of foods without an HSR was higher (IQR = 1070) than that for the groups of foods with an HSR (IQR = 774). A Mann-Whitney U test comparing mean ranks for the products with an HSR and those without found that the groups were not statistically significantly different (U = 21182, *p* = 0.395). The proportions of low, medium and high ED products within the groups of foods without an HSR and with an HSR are shown in Appendix A. A Chi-square test for independence indicated no significant association between ED category and the presence of an HSR, χ2 (1, *n* = 548) = 2.695, *p* = 0.26, Cramer’s V = 0.07.

### 3.3. Products with a Low or High HSR: GNPD Food Category Distributions and Energy Densities

The breakdown of products across food categories for items with low (HSR < 2.5 stars) and high (HSR ≥ 2.5 stars) HSRs is shown in Table 3. Only the categories Bakery and Breakfast Cereals contained products with a low HSR, and these two food categories combined made up 24% of all products displaying an HSR. The remaining 76% of products, those with a high HSR, were spread across nine food categories, with the majority falling under Snacks (52.6%) and Breakfast Cereals (14.5%), both of which had high median ED. The median ED of products with a low HSR was 1850 kJ/100 g (IQR = 147) compared with 1507 kJ/100 g (IQR = 1005) for products with a high HSR. Although both of these medians represent high ED, statistically, the median ED of products with a high HSR (M = 42.26) was lower than the median of those with a low HSR (M = 76.58; U = 286, *p* < 0.05).

**Figure 1:**
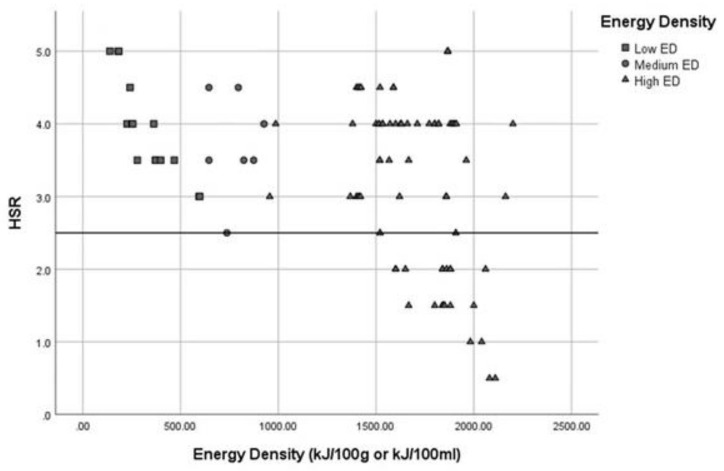
Scatterplot of product Health Star Rating (HSR) by low, medium and high energy density (ED).

The median HSR for all 100 products with an HSR was 3.5 stars (IQR=1.5), with a range of 0.5 to 5 stars. A total of 16 of the 100 products with an HSR were from the low ED category, and all scored a high HSR (median 4 stars (IQR = 1.4); range 3 to 5 stars). All seven of the products in the medium ED category scored a high HSR (median 3.5 stars (IQR = 1); range 2.5 to 4.5 stars). The median HSR for the high ED category was also 3.5 stars (IQR=2) but with the full range of 0.5 to 5 stars represented. Figure 1 shows the scatterplot of HSRs by low, medium and high ED categories. Among the 77 products from the high ED category, only 24 (31%) scored a low HSR. However, 53 (69%) of the products, categorised as high ED, also scored a high HSR, with the majority of these categorised as Snacks. The breakdown of all 100 products by ED and HSR categories is shown in Appendix B.

### 3.4. Core vs. Discretionary Products: Category Distributions, Energy Densities and HSRs

The breakdown of products with HSRs as core or discretionary foods across food categories is shown in Table 4. Overall, 30% of products displaying an HSR were classified as core and 70% as discretionary. The categories Bakery and Snacks combined accounted for 81.5% of all discretionary products. The median ED of core products was 971 kJ/100 g (IQR = 1164) compared with 1800 kJ/100 g (IQR = 355) for discretionary products, with both medians in the high ED range. The EDs of core products (M = 28.72) were significantly lower than those of discretionary products (M = 59.84; U = 1703.5, *p* < 0.05). The distribution of core and discretionary products across the three categories of ED is shown in Appendix C.

The median HSR for core products was 4 stars (IQR = 0.5) and ranged from 2 to 5 stars. For discretionary products, the median was 3.5 stars (IQR = 2.0), with the full range of 0.5 to 5.0 stars. Only 3% of core foods displayed a low HSR and 97% a high HSR. On the other hand, only 33% of discretionary foods displayed a low HSR, whereas the majority (67%) of discretionary foods displayed a high HSR. The distribution of core and discretionary products across the two categories of HSR is shown in Appendix D.

## 4. Discussion

Between June 2014 and June 2018, the majority of new food products targeted to Australian children had high ED. Less than 20% of products displayed an HSR, and the HSR system did not consistently distinguish between low ED and high ED products. About three-quarters of products with an HSR were categorised as having a high HSR, and the majority of products with an HSR (70%) were categorised as discretionary foods.

These findings are consistent with previous Australian and New Zealand research, which found that the majority of food products available for sale [34] and directed at children [35] were considered ‘less healthy’ using nutrient profiling criteria. Additionally, several population-based surveys have found that Australian children regularly consume high ED, nutrient-poor foods [17,18,19]. While it is important to encourage children to meet dietary recommendations and energy needs through healthful food intake and limited intake of high ED, nutrient-poor foods, additional strategies targeting the retail food market have the potential to assist in moderating children’s dietary energy density and energy intake. For example, a recent study showed that total and saturated fat reformulation of some UK supermarket bakery items (cakes and biscuits) could result in substantial reductions in product energy density [36]. In the present study, a large proportion (18%) of the products that entered the retail food market during the four years of interest were bakery items, suggesting a large segment of the market that could also be reformulated in Australia. While food reformulation of processed foods is potentially useful to reduce the energy density of the food supply, it must not be used as a way to increase the perceived healthfulness of discretionary processed foods.

An HSR was displayed on 18.2% of products examined in this study. This result is higher than that obtained in a study by Lawrence et al., who found that 10.5% of new products (using Mintel’s GNPD) released between 27th June 2014 and 27th June 2017 displayed an HSR, and a study by Dickie et al. using the same database but for the time period 6 June 2014–30 June 2019, who found an HSR on 17.6% of products [37,38]. Differences in the database dates used, target sample and/or the product sample size could explain these differing proportions of foods displaying an HSR. Bakery and Snacks categories were the most prevalent products displaying an HSR, as also found by Lawrence et al. and Dickie et al. [37,38]. The food categories in this study that did not have any products displaying an HSR were mostly discretionary foods, for example, Chocolate Confectionary and Sugar and Gum Confectionery. As the HSR system is currently voluntary, manufacturers can selectively apply the HSR to products receiving higher ratings. For example, Shahid et al. reported that for a number of manufacturers, there was a 1.9 to 2.5-star difference between mean HSR displayed on their products compared with their other products that did not display the HSR [23]. This is also supported by the finding that just over three-quarters of products in this study had an HSR ≥ 2.5 stars with a median across the whole sample at an HSR of 3.5 stars.

Despite the voluntary nature of the HSR and the propensity of manufacturers to apply the HSR to higher scoring foods, there was no significant difference between numbers of products with and without HSRs in each of the three ED categories. It could be hypothesised that if the HSR system was better aligned with ED, there would be a greater proportion of products with an HSR in the low ED category [23]. This is the first study to look at ED and HSRs, so it is not possible to compare this finding to the existing literature.

Among the foods displaying an HSR, all low and medium ED foods displayed a high HSR, as would be expected. However, only 31% of high ED foods displayed a low HSR, with the remaining 69% displaying a high HSR. Some high ED products may deserve high HSR. For example, The Happy Snack Company’s Roasted Fav-va Beans in four different flavours have an ED of 1867 kJ/100 g, yet score highly on the HSR algorithm for being high in protein and fibre and containing more than 80% legume. However, there are also products that are clearly discretionary, such as Messy Monkey Strawberry and Apple Snack Bars by Freedom Foods. This snack item has 4.5 stars, yet has an ED of 1410 kJ/100 g, is one-third sugar, and contains mostly dried fruit, which is recommended as occasional by the Australian Dietary Guidelines [39]. If the HSR was classifying foods correctly on the basis of ED, then we would expect a much lower percentage of high ED foods displaying a high HSR. This further adds to the body of literature, demonstrating the shortcomings of the HSR system [23,37,38,40,41,42,43], and shows that it does not consistently discriminate between levels of ED, especially when considering high ED foods. The median ED of products with a high HSR was 1507 kJ/100 g, well above the cut-off (950 kJ/100 g), signifying the beginning of the high ED range [31].

The classification of food products into core and discretionary groups seemed to align more accurately with the ED categories, with increasing proportions of discretionary foods in each increasing ED category. This supports previous studies that have shown that high ED is associated with discretionary foods [9,13,15,44]. The results relating to core foods (only 3% displaying a low HSR) indicated good concordance between core foods and HSR. However, the same could not be concluded with regard to discretionary foods, with 67% displaying a high HSR. Consistent with this study, Lawrence et al. found that 57% of discretionary foods had an HSR ≥ 2.5, and Pulker et al. found that 55% of ultra-processed foods carried HSRs ≥ 3 [27,37]. These findings are concerning in that they show that the HSR is likely to have the opposite effect to what Hawkes et al. posit the role of front-of-pack nutrition labels should be—to decrease the perceived healthiness of discretionary products rather than increase the perceived healthiness of healthy products [45]. By not accurately discriminating amongst discretionary and high ED foods, the HSR is effectively allowing these foods to be perceived as healthier than they actually are.

Several studies in Australia and New Zealand have found that consumers prefer HSRs over other packaging labels, such as nutrition information panels or daily guide, although product visuals (for example, artificial or natural looking food, pictures of fresh fruit, images of sport, etc.) were found to be the foremost influence on choice [46,47,48,49]. Hamlin et al. performed a longitudinal study on the effectiveness of the HSR and, despite heavy advertising campaigns for the HSR system in New Zealand, found it to be ineffective at influencing the customer in their choice between products in a food category [50]. Likewise, Ares et al. found the HSR to be less effective than Nutri-score and a warning symbol in catching attention, healthiness perception and intention to purchase (Comparison of three systems) [51]. An international comparison of a number of different front-of-pack nutrition labels found that most increased consumer ability to rank food healthfulness but that colour coded varieties, such as Nutri-score and traffic lights, were more beneficial than the HSR [52].

In light of the continuing support for the expansion of HSRs, it is imperative that the system provides appropriate guidance for shoppers in making food choices in line with the Australian Dietary Guidelines [39]. We have shown here that when choosing between two products with HSRs, selecting the food with the greater number of health stars will not always be the “healthier” or lowest ED choice [42]. With most new foods marketed to children categorised as high ED and the majority of those with an HSR considered discretionary, consumers need a more consistent measure of healthiness.

This is the first study to examine new food products targeted at children entering the Australian retail food market and assess their ED and discretionary or core grouping with their HSR. The Mintel GNPD is comprehensive, up-to-date and well suited to this study and its aims, as it focuses on new product activity. This is particularly relevant as new food products represent ways in which manufacturers have responded to the introduction of the HSR system. It should also be noted that the GNPD does not reflect a product’s market share, only its existence, and so the product’s pervasiveness in the diets of Australian children is unclear.

It is difficult to keep up with innovations and developments in food items, making it difficult for the Australian Bureau of Statistics Discretionary Food List to accurately distinguish between discretionary and core foods. Errors may have occurred in classifying the 100 products displaying an HSR into discretionary or core categories. However, to reduce the possibility of error, the coding into categories was checked by both co-researchers.

It would be of benefit to extend the work of the current study to cover all food products on the Australian market targeted to children and not just new foods. This study raises questions regarding the three-way relationship between ED, discretionary foods and HSRs. Future research that combines the analysis of these three measures using a larger sample of foods would further the knowledge in this area. The present study weighted all food products equally and not by market share. Research to analyse EDs of food products and adjust their impact using their prevalence in the supermarket would help deepen the understanding around foods available to children. It would also be of benefit to undertake similar research for seasonal products, that is, analysing their availability in the existing market and their market share, as well as studies to measure the impact of seasonal foods on children’s diets. Further research is needed into the effectiveness of the HSR system on whether it is meeting its objectives for consumers at the point of sale and resulting in the purchasing of healthier food products.

The Australian Government acknowledges the need to take action against obesity in children by improving the food environment and, therefore, individual diets through the introduction of initiatives, such as the HSR System [22]. The Australian food industry is also making attempts to improve the food environment by introducing voluntary guidelines to reduce the levels of saturated fat, sodium and energy in foods targeted to children. However, these initiatives by the food industry and the Government to get children eating healthier foods will likely have difficulty translating into positive results while they remain voluntary and unenforced [53].

## 5. Conclusions

A high proportion of new food products targeted to children is of high ED, and the HSR of these foods, when displayed, does not consistently discriminate between levels of ED or between core and discretionary foods. Most new products for children that display HSR are discretionary foods, which are likely contributing to lower diet quality and excess EI. There exist potential opportunities (prompted by food manufacturers wanting to achieve higher HSRs) to reduce the ED of some of these foods to help curb excess EI and improve diet quality. The results of this study support the need to advocate for a food policy change that will result in lower ED foods and improvements to the accuracy and consistency of the HSR system, with the aim to improve the diet quality of Australian children and reduce rates of childhood obesity.

## Figures and Tables

**Table 1:** Numbers of products in each Global New Products Database (GNPD) food category classified as low, medium and high energy density (ED) and median ED for each GNPD food category.

GNPD Food Category ^1^	*n* (%) ^2^				kJ/100 g or kJ/100 mL			
	Low ED (<630 kJ/100 g)	Medium ED (630–950 kJ/100 g)	High ED (>950 kJ/100 g)	Total Products	Median	IQR ^3^	Minimum	Maximum
Baby Food	8 (1.5)	1 (0.2)	10 (1.8)	19 (3.5)	1467	1477	273	2380
Bakery	0 (0)	2 (0.4)	100 (18.2)	102 (18.6)	1845	291	871	2180
Breakfast Cereals	1 (0.2)	0 (0)	29 (5.3)	30 (5.5)	1586	94	411	1711
Chocolate Confectionery	0 (0)	0 (0)	30 (5.5)	30 (5.5)	2265	120	1867	2360
Dairy	37 (6.8)	0 (0)	15 (2.7)	52 (9.5)	377	853	290	1790
Desserts and Ice Cream	41 (7.5)	8 (1.5)	4 (0.7)	53 (9.7)	409	334	6	1300
Fruit and Vegetables	3 (0.5)	0 (0)	0 (0)	3 (0.5)	138	-	60	242
Meals and Meal Centres	7 (1.3)	3 (0.5)	1 (0.2)	11 (2.0)	468	359	260	956
Processed Fish, Meat and Egg Products	0 (0)	9 (1.6)	1 (0.2)	10 (1.8)	806	142	724	975
Savoury Spreads	0 (0)	0 (0)	1 (0.2)	1 (0.2)	1092	-	1092	1092
Side Dishes	0 (0)	1 (0.2)	1 (0.2)	2 (0.4)	1143	-	795	1490
Snacks	20 (3.6)	3 (0.5)	145 (26.5)	168 (30.7)	1613	457	227	2556
Sugar and Gum Confectionery	0 (0)	1 (0.2)	65 (11.9)	66 (12.0)	1462	203	731	2130
Sweet Spreads	0 (0)	0 (0)	1 (0.2)	1 (0.2)	1413	-	1413	1413
Total	117 (21.4)	28 (5.1)	403 (73.5)	548	1514	1006	6	2556

^1^ No products found in the Sauces and Seasonings, Soup and Sweeteners and Sugar food categories. ^2^ Percentages may not equal 100 due to rounding. ^3^ Indicates not possible to calculate due to a low number of products in the category. IQR, interquartile range.

**Table 2:** The energy density of products without a Health Star Rating (HSR) and with an HSR by Global New Products Database (GNPD) food category.

GNPD Food Category ^1^	Products without HSR					Products with HSR				
	*n* (%) ^2^	kJ/100 g or kJ/100 mL				*n* (%) ^2^	kJ/100 g or kJ/100 mL			
		Median	IQR ^3^	Minimum	Maximum		Median	IQR ^3^	Minimum	Maximum
Baby Food	19 (4.2)	1467	1477	273	2380	0 (0)	−	−	−	−
Bakery	78 (17.4)	1837	453	871	2180	24 (24.0)	1870	137	986	2110
Breakfast Cereals	14 (3.1)	1575	94	411	1668	16 (16.0)	1586	107	1400	1711
Chocolate Confectionery	30 (6.7)	2265	120	1867	2360	0 (0)	−	−	−	−
Dairy	48 (10.7)	379	970	290	1790	4 (4.0)	372	27	363	399
Desserts and Ice Cream	47 (10.5)	409	335	6	1300	6 (6.0)	389	451	181	737
Fruit and Vegetables	1 (0.2)	60	−	60	60	2 (2.0)	190	-	138	242
Meals and Meal Centres	5 (1.1)	290	113	260	474	6 (6.0)	645	474	279	956
Processed Fish, Meat and Egg Products	9 (2.0)	788	130	724	975	1 (1.0)	927	−	927	927
Savoury Spreads	1 (0.2)	1092	−	1092	1092	0 (0)	−	−	−	−
Side Dishes	1 (0.2)	1490	−	1490	1490	1 (1.0)	795	−	795	795
Snacks	128 (28.6)	1613	467	229	2556	40 (40.0)	1607	454	227	2200
Sugar and Gum Confectionery	66 (14.7)	1462	203	731	2130	0 (0)	−	−	−	−
Sweet Spreads	1 (0.2)	1413	−	1413	1413	0 (0)	−	−	−	−
Total	448	1490	1070	6	2556	100	1594	774	138	2200

^1^ No products found in the Sauces and Seasonings, Soup and Sweeteners and Sugar food categories. ^2^ Percentages may not equal 100 due to rounding. ^3^ Indicates not possible to calculate due to a low number of products in the category.

**Table 3:** The energy density of products with a Health Star Rating (HSR) <2.5 and with an HSR≥2.5 by Global New Products Database (GNPD) food category.

GNPD Food Category ^1^	Products with HSR < 2.5					Products with HSR ≥ 2.5				
	*n* (%)	kJ/100 g or kJ/100 mL				*n* (%)	kJ/100 g or kJ/100 mL			
		Median	IQR	Minimum	Maximum		Median	IQR ^2^	Minimum	Maximum
Bakery	19 (79.2)	1880	160	1800	2110	5 (6.6)	1860	677	986	1961
Breakfast Cereals	5 (20.8)	1600	58	1600	1667	11 (14.5)	1533	178	1400	1711
Dairy	0 (0)	−	−	−	−	4 (5.3)	372	27	363	399
Desserts and Ice Cream	0 (0)	−	−	−	−	6 (7.9)	389	451	181	737
Fruit and Vegetables	0 (0)	−	−	−	−	2 (2.6)	190	−	138	242
Meals and Meal Centers	0 (0)	−	−	−	−	6 (7.9)	645	474	279	956
Processed Fish, Meat and Egg Products	0 (0)	−	−	−	−	1 (1.3)	927	−	927	927
Side Dishes	0 (0)	−	−	−	−	1 (1.3)	795	-	795	795
Snacks	0 (0)	−	−	−	−	40 (52.6)	1607	454	227	2200
Total	24 (100)	1850	147	1600	2110	76 (100)	1507	1005	138	2200

^1^ No products found in the Baby Food, Chocolate Confectionery, Sauces and Seasonings, Savoury Spreads, Soup, Sugar and Gum Confectionery, Sweet Spreads and Sweeteners and Sugar food categories. ^2^ Indicates not possible to calculate due to a low number of products in the category.

**Table 4:** The energy density of the core and discretionary products displaying the Health Star Ratings (HSRs) by Global New Products Database (GNPD) food category.

GNPD Food Category ^1^	Core					Discretionary				
	*n* (%)	kJ/100 g or kJ/100 mL				*n* (%)	kJ/100 g or kJ/100 mL			
		Median	IQR ^2^	Minimum	Maximum		Median	IQR ^2^	Minimum	Maximum
Bakery	1 (3.3)	986	−	986	986	23 (32.9)	1880	142	1520	2110
Breakfast Cereals	11 (36.7)	1533	198	1400	1711	5 (7.1)	1600	50	1567	1667
Dairy	4 (13.3)	372	27	363	399	0 (0)	−	−	−	−
Desserts and Ice Cream	0 (0)	−	−	−	−	6 (8.6)	389	451	181	737
Fruit and Vegetables	2 (6.7)	190	−	138	242	0 (0)	−	−	−	−
Meals and Meal Centers	6 (20.0)	645	474	279	956	0 (0)	−	−	-	−
Processed Fish, Meat and Egg Products	0 (0)	−	−	−	−	1 (1.4)	927	−	927	927
Side Dishes	0 (0)	−	−	−	−	1 (1.4)	795	−	795	795
Snacks	6 (20.0)	828	1296	227	1908	34 (48.6)	1645	378	823	2200
Total	30 (100)	971	1164	138	1908	70 (100)	1800	355	181	2200

^1^ No products found in the Baby Food, Chocolate Confectionery, Sauces and Seasonings, Savoury Spreads, Soup, Sugar and Gum Confectionery, Sweet Spreads and Sweeteners and Sugar food categories. ^2^ Indicates not possible to calculate due to a low number of products in the category.

**Figure A1:**
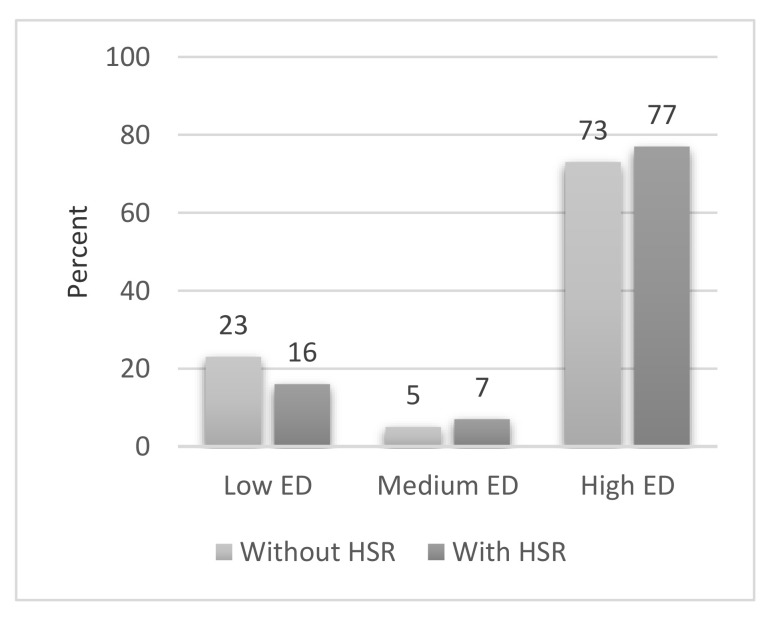
Proportions of low, medium and high energy density (ED) products without and with a Health Star Rating (HSR).

**Figure A2:**
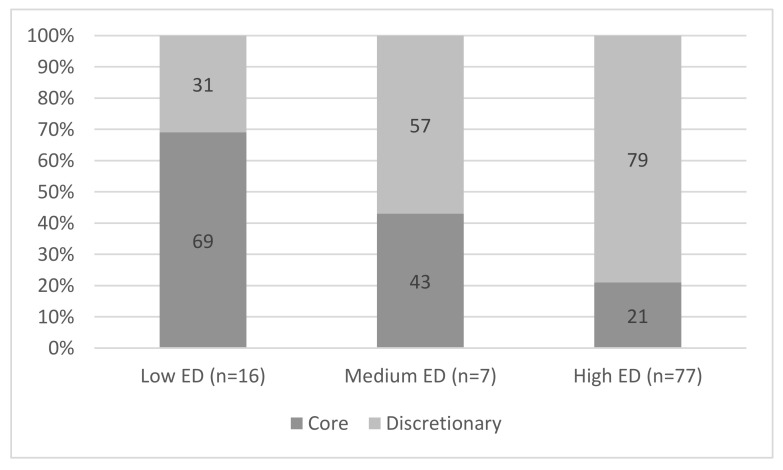
Core and discretionary products displaying the Health Star Ratings (HSRs) by energy density (ED) category.

**Figure A3:**
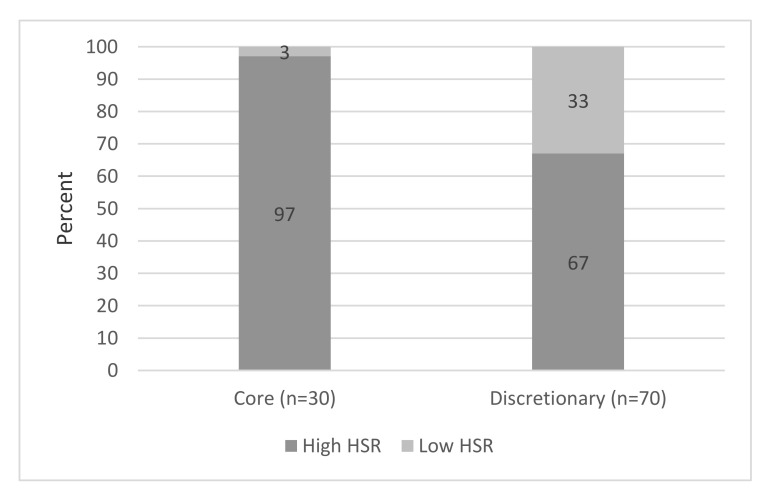
Core and discretionary foods by Health Star Rating (HSR) category (*n* = 100).

## Appendix B

**Table A1:** Number of products by energy density (ED) and Health Star Rating (HSR) categories.

GNPD Food Category ^1^	Energy Density						Total
	Low ED		Medium ED		High ED		
	Low HSR	High HSR	Low HSR	High HSR	Low HSR	High HSR	
Bakery	0	0	0	0	19	5	24
Breakfast Cereals	0	0	0	0	5	11	16
Dairy	0	4	0	0	0	0	4
Desserts and Ice Cream	0	5	0	1	0	0	6
Fruit and Vegetables	0	2	0	0	0	0	2
Meals and Meal Centers	0	2	0	3	0	1	6
Processed Fish, Meat and Egg Products	0	0	0	1	0	0	1
Side Dishes	0	0	0	1	0	0	1
Snacks	0	3	0	1	0	36	40
Totals	0	16	0	7	24	53	100

^1^ No products found in the Baby Food, Chocolate Confectionery, Sauces and Seasonings, Savoury Spreads, Soup, Sugar and Gum Confectionery, Sweet Spreads and Sweeteners and Sugar food categories.
